# Acute stent thrombosis in a sirolimus eluting stent after wasp sting causing acute myocardial infarction: a case report

**DOI:** 10.4076/1757-1626-2-7800

**Published:** 2009-08-12

**Authors:** Martin Greif, Tilmann Pohl, Nico Oversohl, Christopher Reithmann, Gerhard Steinbeck, Alexander Becker

**Affiliations:** Department of Cardiology, Ludwig-Maximilians-UniversityMunich, Germany Marchioninistrasse 1581377 MunichGermany

## Abstract

**Introduction:**

Hymenoptera venoms contain thrombogenic substances that might be responsible for cardiovascular events independent of anaphylactic reactions.

**Case presentation:**

We report a 55-year-old man who experienced an acute ST-elevation myocardial infarction after wasp sting. The patient presented without signs of anaphylaxis or shock. The coronary angiography showed an acute stent thrombosis of the right coronary artery. Percutanous coronary intervention was performed immediately and this is an example for a cardiovascular complication associated with a hymenoptera sting, since the vasoactive, inflammatory, and thrombogenic substances of hymenoptera venoms potentially cause stent thrombosis and myocardial ischemia. To the best of our knowledge this is the first report of acute stent thrombosis in a sirolimus-eluting stent following hymenoptera sting.

**Conclusion:**

Stent thrombosis is a possible complication after wasp sting induced by thrombogenic substances of the hymenoptera venom.

## Introduction

Hymenoptera venoms contain thrombogenic substances that might be responsible for cardiovascular events, especially in patients with coronary artery disease. Independent of anaphylactic reactions phospholipase A_1_ of the Hymenoptera venoms can induce thrombogenic reactions the might lead to acute arterial thrombosis.

## Case presentation

A 55-year-old Caucasian German man with known ischemic heart disease presented in our emergency room, with an acute inferior myocardial ST-elevation infarction. Three years before stenoses of the proximal left anterior descending and the proximal right coronary artery were treated by PCI and implantation of two bare metal stents, followed by PCI and implantation of a sirolimus-eluting stent (Cypher®) of an In-stent restenosis in the right coronary artery one year later. Afterwards no relevant cardiovascular events or hospitalizations occurred until the current admission. Clopidogrel (75 mg/d) was withdrawn 12 months after implantation of the sirolimus-eluting stent, while aspirin (100 mg/d) was continued. Known and treated cardiovascular risk factors were hypertension, hypercholesterolemia and diabetes mellitus.

**Figure 1.**
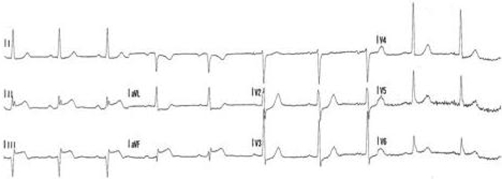
ECG registered by the emergency physician with ST-segment elevation in the inferior leads.

As hobby beekeeper the patient was used to bee stings and never reported of any related symptoms, except local reactions. One hour before admission he was stung by 5 wasps. Just a few minutes after being stung he started complaining of chest pain and called an emergency physician. ECG revealed an acute myocardial infarction with inferior ST-segment-elevation (Figure 1), blood pressure was 140/85 mmHg, heart rate was 59/minute. The patient showed no signs of anaphylaxis or shock, neither any other complains. Aspirin (500 mg) and heparin (5000 IU) had been given by the emergency physician, chest pain improved after treatment with morphine.

**Figure 2.**
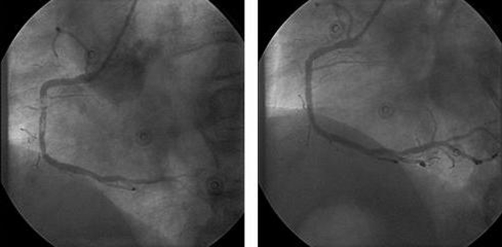
Acute stent thrombosis of the right coronary artery before **(Left)** and after PCI **(Right)**.

Immediately after admission to the emergency room, coronary angiogram was performed showing a thrombosis in the two year old sirolimus-eluting stent of the right coronary artery occluding the vessel sub-totally. PCI was successfully performed with good angiographic result (Figure 2). Therapy with tirofibane (Aggrastat®, MSD, Germany) was started during intervention in a weight adjusted dose and had been continued for 24 hours. 600 mg clopidogrel were admitted following PCI, oral therapy was continued with 75 mg/day. Post interventional clinical course was uneventful, the patient recovered quickly, echocardiography showed left ventricular function within normal limits (fractional shortening 34%) without any regional wall-motion disturbances. The patient was discharged 6 days after myocardial infarction on a regime of aspirin, clopidogrel, bisoprolol, ramipril and simvastatin.

## Discussion

Myocardial infarction following hymenoptera stings (e.g. bees, wasps and hornets) is rare but there are some cases reported in the literature [1-5]. However to the best of our knowledge this is the first report of acute stent thrombosis in a drug-eluting stent caused by a wasp sting leading to myocardial infarction.

Despite from local cutaneous manifestations or generalized anaphylaxis, hymenopterans stings can cause a variety of unusual reactions. There have been reports of neurological reactions such as myasthenia gravis, peripheral neuritis and Guillain-Barré syndrome. Diffuse alveolar hemorrhage, acute renal failure, rhabdomyolysis, thrombocytopenic purpura and vasculitis are other pathologies presumed to be related to insect stings [6].

Myocardial infarction after bites of hymenopterans is mostly reported to be preceded by an allergic reaction, sometimes with angiographic evidence of undamaged coronary arteries [4]. Pathophysiological determinant seems to be related to the chemical composition of hymenopterans venom, basically containing vasoactive, inflammatory and thrombogenic substances (e.g. histamine, serotonin, dopamine, leukotrienes, thromboxanes and bradykinin) and potentially allergenic proteins (e.g. phospholipases, hyaluronidases, acid phosphatases, and mellitin) able to create vasospasm and/ or coronary thrombosis [7,8]. Histamine and Serotonin are stimulating endogenous secretion of adrenalin and noradrenalin. The vasoactive mediators (e.g. histamine, serotonin, dopamine, noradrenaline) can lead also to myocardial ischemia either by hypotension or by increased myocardial oxygen demand due to direct inotropic or chronotropic effects, especially in patients with known coronary artery disease [2]. Many mediators influence potentially significant actions on coagulation, favouring platelet aggregation and thrombosis [9]. Yang *et al.* recently described a protein, magnifin, which was purified from the venom of a wasp *Vespa magnifica (Smith)*, containing phospholipase-like activity and inducing platelet aggregation and thrombosis *in vivo* [10]. Chen *et al.* reported a case of thrombosis of the descending aorta and cerebral infarction after massive wasp stings [11].

In the cases reported, the causal relationship between hymenoptera sting and myocardial ischemia was sometimes not very clear. Some patients had signs of anaphylactic shock and therefore were treated with adrenaline. On one hand hypotension cased by anaphylaxis can lead to myocardial hypoperfusion, on the other hand adrenaline itself has thrombogenic effects and is able to provocate vasoconstriction [4], contributing to aggravation of myocardial ischemia. Korantzopoulos *et al.* reported of an acute ST-elevation myocardial infarction in the context of an anaphylactic reaction caused by a European hornet sting. In presence of ST-elevations at presentation the patient was not treated with adrenaline. In this patient exogen adrenalin did not contribute to the development of the myocardial infarction, but hypotension due the anaphylactic shock may have contributed to the development or the worsening of myocardial infarction [1].

In our patient no signs of anaphylaxis or shock were present. First symptoms of chest pain occurred a few minutes after the wasp stings. Immediately afterwards the patient called the emergency physician. Initial ECG revealed ST-elevations in leads II, III and aVF, while blood pressure was within normal range (140/85 mmHg). Considering the short time interval between the wasp attack and the beginning of chest pain, it is reasonable to assume, that the wasp venom caused acute stent thrombosis followed by myocardial infarction.

It is notable that our patient as beekeeper was used to bee stings and has been stung by bees many times without any symptoms except for local reactions. As bee and wasp venoms are highly complicated mixtures of pharmacologically or biochemically active agents, it remains unclear which components of the wasp venom differing from bee venom induced the acute stent thrombosis. Interestingly wasp venom contains more phospholipase A_1_ as phospholipase A_2_ in contrast to bee venom [12]. This and the finding of Yang *et al.* that phospholipase A_1_ isolated from wasp venom inducing platelet aggregation and thrombosis in vivo [10], could be crucial for the distinct reaction of our patient to different hymenoptera venoms.

## Conclusion

Stent thrombosis is a possible complication after wasp sting induced by thrombogenic substances as for example phospholipase A_1_ of the hymenoptera venom.

## Abbreviations

ECG: electrocardiogram
PCI: percutanous coronary intervention
